# A Derivative Approach to Permittivity Extraction from Free-Space S-Parameters Measurements

**DOI:** 10.3390/s26154832

**Published:** 2026-07-31

**Authors:** Giancarlo Bartolucci, Giovanni Capoccia, Romolo Marcelli, Emanuela Proietti

**Affiliations:** 1 Department of Electronic Engineering, University of Roma Tor Vergata, 00133 Rome, Italy; bartoluc@uniroma2.it; 2 CNR-Institute for Microelectronics and Microsystems, 00133 Rome, Italy; giovanni.capoccia@cnr.it (G.C.); romolo.marcelli@cnr.it (R.M.)

**Keywords:** free-space measurement, complex permittivity, derivative method

## Abstract

The contactless characterization of dielectric materials at microwave frequencies in terms of permittivity is a long-standing need in materials science and electromagnetic engineering, and free-space techniques are attractive because they require no sample preparation and operate over broadbands. Conventional Nicolson–Ross–Weir inversion and its iterative variants either suffer from the half-wavelength phase-ambiguity problem or require a reliable initial guess to converge; this work introduces a method that avoids both limitations. The permittivity is retrieved from the frequency derivative of the transmission coefficient T, extracted from the measured S-parameters. As the formulation involves only real-valued quantities, it is intrinsically immune to phase ambiguity and needs no initial estimate. Measurements used two patch antennas separated by 15 cm and a Keysight P9375A Vector Network Analyzer, with the derivative computed through a local polynomial fit. After confirming the negligible-loss regime, the real permittivity was extracted for PMMA, Polymethyl methacrylate (PMMA, ε_r_ ≈ 3.31), dry sand (ε_r_ ≈ 3.59), and mineral oil (ε_r_ ≈ 1.85), all of which are consistent with the literature data.

## 1. Introduction

The contactless, non-destructive characterization of dielectric materials at microwave frequencies in terms of permittivity is a well-established requirement in materials science, industrial inspection, and electromagnetic engineering. Free-space measurement techniques, in which a planar sample is illuminated by a normally incident plane wave and the complex reflection and transmission coefficients (S-parameters) are acquired by a Vector Network Analyzer (VNA), are particularly attractive because they require no sample preparation, impose no dimensional constraints, and operate over broad frequency bands.

The foundations of free-space permittivity measurement were laid by Ghodgaonkar and Varadan in two seminal papers. In their 1989 work [1], they introduced a system based on spot-focusing horn-lens antennas operating at 14.5–17.5 GHz, using TRL (Thru-Reflect-Line) free-space calibration and time-domain gating to suppress multiple reflections; the complex permittivity was extracted from the metal-backed reflection coefficient S_11_ by a zero-finding algorithm applied to the transmission-line equation. The 1990 extension [2] generalized the method to simultaneous measurement of complex permittivity and complex permeability from both S_11_ and S_21_, extending coverage to the 8.2–40 GHz range. These two contributions established the accuracy benchmarks and the calibration philosophy that underpin virtually all subsequent free-space dielectric measurement systems.

The analytical backbone common to all transmission/reflection (T/R) methods was formalized by Nicolson and Ross [3] and by Weir [4] in the 1970s. In the Nicolson–Ross–Weir (NRW) algorithm, the reflection coefficient Γ and transmission coefficient T of the sample are extracted from S_11_ and S_21_ via closed-form inversion, yielding both ε and μ explicitly. Baker-Jarvis, Vanzura, and Kissick [5] later identified that the NRW solution becomes ill-conditioned at frequencies where the sample thickness equals an integer multiple of the half-wavelength inside the material and proposed robust iterative alternatives that remain stable across the full spectrum.

For environments in which the measurement antennas do not support focused Gaussian beams, simpler horn-based free-space approaches have been demonstrated. Kumar et al. [6] showed that ordinary pyramidal horn antennas without any focusing mechanism can yield accurate complex permittivity values, provided the sample area is large enough, and TRL calibration with time-domain gating is applied. Awang et al. [7] extended this philosophy to transmission and metal-backed methods for measuring double-layer bulk dielectrics and thin-film oxides at 18–26 GHz. Chang et al. [8] further extended free-space measurement to the terahertz band (325–500 GHz), combining a VNA with frequency-extension modules, GRL (Gated-Reflect-Line) calibration, and a Newton iterative algorithm. Qin et al. [9] proposed a variant that uses two reflection measurements to avoid the phase-ambiguity problem entirely. The NRW family of methods has also been applied beyond classical dielectrics: Smith et al. [10] applied the T/R inversion to effective permittivity and permeability of electromagnetic metamaterials.

In a previous contribution [11], we applied a free-space transmission setup with microstrip patch antennas to detect mineral oil aging, retrieving the permittivity from the phase of the transmission coefficient. In that work, the phase-ambiguity problem inherent in the NRW formulation was avoided by an appropriate choice of the sample thickness, keeping the MUT thin enough for the phase unwrapping to remain unambiguous over the measurement band. While effective, this strategy constrains the experiment, since the admissible thickness must be verified a priori for each sample.

Methods based on iterative procedures require an initial guess, whose correct choice is crucial for the algorithm to converge. Using a sample shorter than the wavelength is viable only at moderate frequencies and when the dielectric permittivity is already approximately known. This paper presents a new approach that avoids the multi-value problem and needs no initial estimate, based on the frequency derivative of the transmission coefficient.

The S-parameters are acquired in a broadband free-space configuration, with two patch antennas separated by 15 cm and recorded using a Keysight P9375A Vector Network Analyzer (VNA) (Keysight Technologies, Santa Rosa, CA, USA). Three dielectric materials are characterized: mineral oil, PMMA (plexiglass), and dry sand.

## 2. Materials and Methods

### 2.1. Computation of the Transmission Coefficient T

The transmission coefficient T is extracted from the S-parameters using the Nicolson–Ross–Weir (NRW) inversion procedure in [3,4]. The forward equations model relates the S-parameters of a homogeneous slab to the reflection coefficient Γ and the one-way transmission factor T:

(1) $$T=\frac{S_{11}+S_{12}-\Gamma }{1-\left(S_{11}+S_{12}\right)\cdot \Gamma }$$

(2) $$\Gamma =X\pm \sqrt{X^{2}-1}$$

where:

(3) $$X=\frac{S_{11}^{2}-S_{21}^{2}+1}{2\cdot S_{11}}$$

The sign in (2) is chosen in such a way that it has $\left|\Gamma \right|<1$.

### 2.2. Derivative Method

The resulting T represents the one-way complex transmission through the sample layer alone and can also be written as:

(4) $$T=e^{\gamma }=T_{r}+j\cdot T_{j}$$

With:

(5) $$\gamma =j\cdot k_{0}\cdot \sqrt{\varepsilon_{r}-\varepsilon_{j}}\cdot d=\gamma_{r}-{j\gamma }_{j}$$

$${\mathrm{and}\;k}_{0}=\frac{\omega }{c}$$

where d is the sample thickness, T_r_ is the real part of T, T_j_ is the imaginary part of T, $\gamma_{r}$ is the real part of γ, and $\gamma_{j}$ is the imaginary part of γ.

Differentiating Equation (4) with respect to frequency, and assuming that ε is independent of the radian frequency ω:

(6) $$\frac{\mathrm{dT}}{d\omega }=\frac{{\mathrm{dT}}_{r}}{d\omega }+j\frac{{\mathrm{dT}}_{j}}{d\omega }=\frac{{\mathrm{de}}^{\gamma }}{d\omega }=\left(\frac{{d\gamma }_{r}}{d\omega }+j\frac{{d\gamma }_{j}}{d\omega }\right)\cdot T$$

We define:

(7) $$T_{\mathrm{rd}}=\frac{{\mathrm{dT}}_{r}}{d\omega }$$

(8) $$T_{\mathrm{jd}}=\frac{{\mathrm{dT}}_{j}}{d\omega }$$

(9) $$\gamma_{\mathrm{rd}}=\frac{{d\gamma }_{r}}{d\omega }$$

(10) $$\gamma_{\mathrm{jd}}=\frac{{d\gamma }_{j}}{d\omega }$$

Inserting (7)–(10) into (6), we find:

(11) $$T_{\mathrm{rd}}+{\mathrm{jT}}_{\mathrm{jd}}=\left(\gamma_{\mathrm{rd}}+{j\;\gamma }_{\mathrm{jd}}\right)\left(T_{r}+{\mathrm{jT}}_{j}\right)$$

By equating the real part and the imaginary part, the following system of equations is obtained:

$$\left\{\begin{array}{c} T_{\mathrm{rd}}=T_{r}\gamma_{\mathrm{rd}}-T_{j}\;\gamma_{\mathrm{jd}}\\ T_{\mathrm{jd}}=T_{j}\gamma_{\mathrm{rd}}+T_{r}\gamma_{\mathrm{jd}} \end{array}\right.$$

The unknowns are γrd and γjd. The solution of this system can be written as:

(12) $$\gamma_{\mathrm{rd}}=\frac{T_{r}\cdot T_{\mathrm{rd}}+T_{j}\cdot T_{\mathrm{jd}}}{{\left|T\right|}^{2}}$$

(13) $$\gamma_{\mathrm{jd}}=\frac{T_{r}\cdot T_{\mathrm{jd}}-T_{j}\cdot T_{\mathrm{rd}}}{{\left|T\right|}^{2}}$$

From (5), we find:

(14) $$\frac{d\gamma }{d\omega }=\frac{j}{c}\sqrt{\varepsilon_{r}-{j\varepsilon }_{j}}\cdot d=\gamma_{\mathrm{rd}}+{j\gamma }_{\mathrm{jd}}$$

Taking the square of both sides, we obtain:

(15) $$\varepsilon_{r}=\frac{\gamma_{\mathrm{jd}}^{2}-\gamma_{\mathrm{rd}}^{2}}{d^{2}}\cdot c^{2}$$

(16) $$\varepsilon_{j}=\frac{2\gamma_{\mathrm{rd}}\cdot \gamma_{\mathrm{jd}}}{d^{2}}\cdot c^{2}$$

If losses are negligible, we can assume $\varepsilon_{j}=0$. From (5) and (14), we have:

(17) $$\gamma_{r}=0$$

(18) $$\gamma_{\mathrm{rd}}=0$$

Thus, Equations (15) and (13) provide for the refractive index n:

(19) $$n=\sqrt{\varepsilon_{r}}=\frac{\gamma_{\mathrm{jd}}}{d}\cdot c=\frac{T_{r}\cdot T_{\mathrm{jd}}-T_{j}\cdot T_{\mathrm{rd}}}{d\cdot {\left|T\right|}^{2}}\cdot c$$

Two more expressions can be obtained for n and $\varepsilon_{r}$.

Inserting Equation (18) into Equation (12):

(20) $$T_{r}\cdot T_{\mathrm{rd}}+T_{j}\cdot T_{\mathrm{jd}}=0$$

According to Equation (20), the following two cases must be analyzed:

(21) $$T_{\mathrm{jd}}=-\frac{T_{r}\cdot T_{\mathrm{rd}}}{T_{j}}$$

From (21), (13) and (15), we find:

(22) $$n=\sqrt{\varepsilon_{r}}=\frac{c\cdot T_{\mathrm{rd}}}{d\cdot T_{j}}$$

(23) $$T_{\mathrm{rd}}=-\frac{T_{\mathrm{jd}}\cdot T_{j}}{T_{r}}$$

From (23), (13) and (15), we have:

(24) $$n=\sqrt{\varepsilon_{r}}=\frac{c\cdot T_{\mathrm{jd}}}{d\cdot T_{r}}$$

The main advantage over the free-space methods reviewed above is that the permittivity is derived using |T| as well as the real and imaginary parts of the derivative of T, which are real quantities, and is therefore intrinsically immune to the 2πn phase ambiguity. Unlike the NRW-based inversion [3,4,5] and the horn-based and terahertz implementations that still rely on phase information [6,7,8], it requires neither phase unwrapping nor branch selection nor an initial guess, which makes it particularly suitable for thick or high-permittivity samples.

### 2.3. Experimental Setup

**Figure 1:**
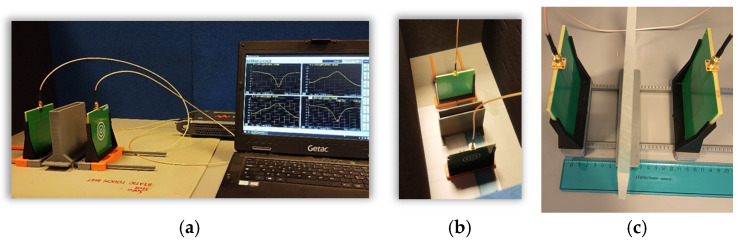
Measurement setup. (**a**) MUT between two patch antennas, VNA and PC, for S-parameter acquisition; (**b**) detail of patch antennas with MUT in between (oil and sand); (**c**) plexiglass.

Measurements are performed in a free-space configuration, with two patch antennas fabricated on a standard FR4 substrate (relative permittivity ε_r_ ≈ 4.3, loss tangent tan δ ≈ 0.02, thickness d = 1.6 mm), with an overall board size of 100 × 100 mm^2^ and a square radiating patch of L = W = 50 mm, placed facing each other at a separation of 15 cm. The material under test (MUT) is a plane-parallel dielectric slab of thickness d, placed mid-way between the antennas (Figure 1). A Vector Network Analyzer (VNA, Keysight P9375A) records the full 4-port S-parameter matrix (S_11_, S_21_, S_12_, S_22_) over the range 1–2 GHz with 1601 frequency points. The materials studied are three dielectric samples: PMMA (8 mm thick), mineral oil (20 mm), and dry sand (20 mm) poured loosely into the container at its natural packing density. In Table 1, all the setup parameters are summarized.

## 3. Results and Discussion

**Figure 2:**
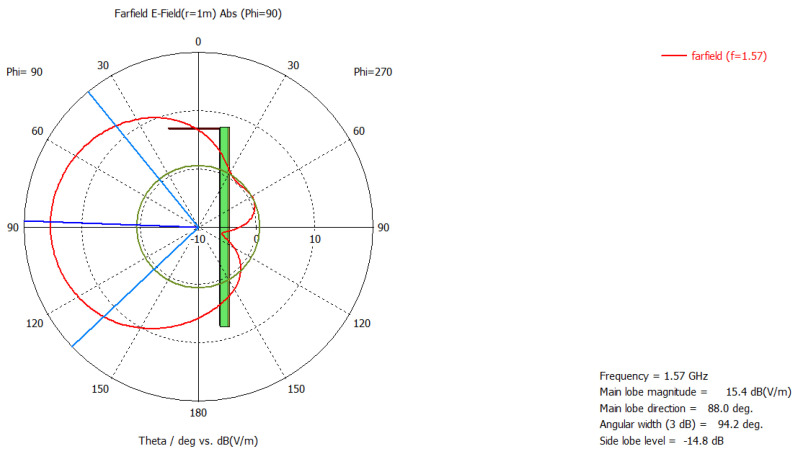
Main lobe direction at 1.57 GHz of the patch antenna.

In this paragraph, electromagnetic simulations and experimental findings will be presented and discussed, applying the analytical approach proposed in the previous sections to obtain the dielectric constant of the material under test. The method assumes the following conditions:

1. Uniform plane wave. The wave impinging on the MUT is assumed to be a uniform plane wave. In practice, the antenna aperture produces a beam of finite width; the approximation holds when the transverse dimensions of the MUT are large enough to intercept the main portion of the antenna beam, keeping edge-diffraction effects negligible, and when the sample lies outside the reactive near-field region of the antenna. Since the patch is electrically small at the operating frequency (L = W = 5 cm, λ ≈ 19 cm at 1.57 GHz), the outer boundary of the reactive near field can be taken as R ≈ λ/2π ≈ 3 cm [12]; the sample, placed at R = 7.5 cm from each antenna, is therefore well outside this region. The far-field simulation of the patch antenna at 1.57 GHz, performed with the commercial software CST Studio Suite 2022, yields a main-lobe direction of 88° (Figure 2) and a −3 dB angular width of 94.2°. At the sample distance R = 7.5 cm, this corresponds to a transverse beam width $W=2R\sin\frac{\Delta \theta }{2}\approx 11\;\mathrm{cm}$, comparable to the 10 cm lateral size of the sample, so that the MUT intercepts the central, most energetic part of the beam.
2. Homogeneous, isotropic, parallel-plane sample. Surface roughness, thickness non-uniformity, or tilt of the MUT with respect to the antenna axis introduce systematic errors that cannot be corrected in post-processing. Sample alignment and accurate thickness measurement are, therefore, among the main sources of uncertainty.
3. Non-dispersive permittivity. Equation (14) is derived assuming that ε is constant with frequency. For weakly dispersive materials, the approximation is valid; for strongly dispersive media, the derivative of ε_r_ and ε_j_ should be included.

**Figure 3:**
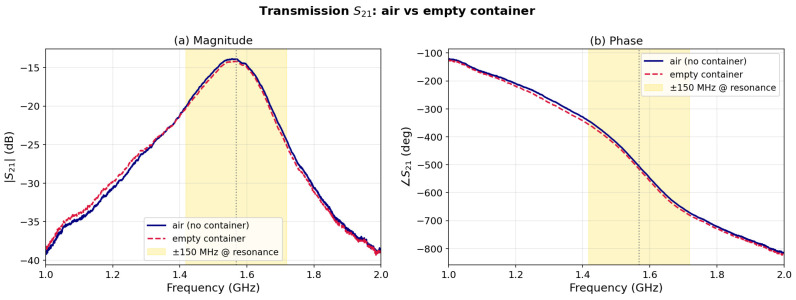
Transmission coefficient S_21_ measured in free space (air, no container) and with the empty container (LxW = 10 × 10 cm^2^, d = 20 mm and wall thickness 2 mm) in the measurement path: (**a**) magnitude and (**b**) phase. The dash line represents the half-bandwidth of ±150 MHz.

A preliminary measurement confirmed that the empty container does not affect the transmission: comparing the free-space response with that of the empty holder, the difference in |S_21_| around the antenna resonance is on average about 0.3 dB (below 4% in relative terms), i.e., within the measurement repeatability (Figure 3). The contribution of the thin container walls is therefore negligible, and the measured transmission can be attributed solely to the MUT.

### 3.1. Numerical Implementation: Polynomial Fitting

Since the transmission parameter T is measured at discrete frequency points, whereas the method requires its frequency derivative, T(f) must be reconstructed as a smooth, continuous function before it can be differentiated. Re(T) and Im(T) are therefore approximated by a polynomial curve, and the derivative is obtained analytically from its coefficients.

**Figure 4:**
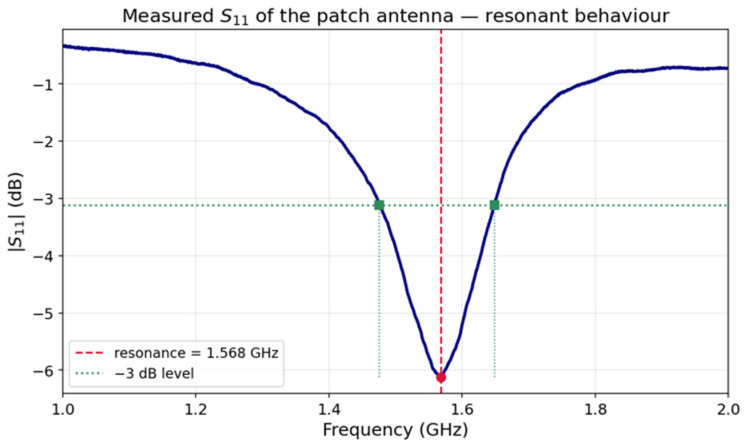
Measured reflection coefficient |S_11_| of the patch antenna, showing its resonant behavior with a minimum at 1.568 GHz. The green markers indicate the frequencies at which |S_11_| rises by 3 dB above the resonance minimum, giving a qualitative measure of the width of the resonance.

A first, natural choice would be to fit a single high-degree polynomial (degree 16) over the whole measured 1–2 GHz band. This global approach, however, must be discarded for two reasons. First, the two patch antennas are intrinsically narrowband: as shown by the measured S_11_ (Figure 4), the antennas are matched only within a limited interval centered on their resonance at approximately 1.57 GHz.

Away from this interval, the transmitted signal drops, and the variation of T(f) is dominated by the frequency-dependent gain and phase response of the antennas rather than by the material, so a polynomial fitted over the whole band encodes the antenna dispersion alongside the material dispersion, and the derivative is contaminated accordingly.

Second, high-degree polynomials fitted to equally spaced data exhibit large spurious oscillations near the endpoints (Runge’s phenomenon [13]), which further corrupt the derivative at the band edges.

**Figure 5:**
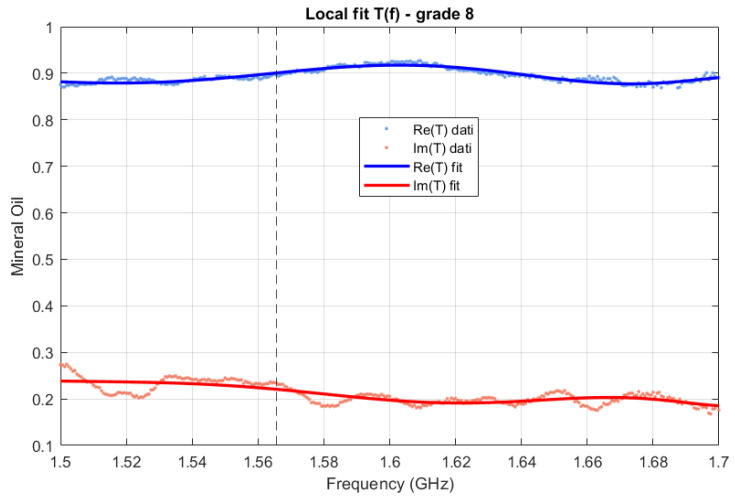
Transmission parameter T (real and imaginary parts): experimental data and degree-8 polynomial fit within the ±150 MHz window centered on the S11 resonance (~1.565 GHz). The material is mineral oil, with a thickness d = 20 mm.

Both problems point to the same conclusion: the extraction must be confined to a narrow window around the antenna resonance, where the signal is strong, and the variation of T(f) is governed primarily by the dielectric response of the MUT. Accordingly, the adopted approach fits a lower-degree polynomial (degree 8) exclusively within a ±150 MHz window centered on the S_11_ resonance. The half-bandwidth of ±150 MHz was chosen as a compromise between keeping the fit close to the antenna resonance, where the transmitted signal is strong and governed by the material response, and retaining enough measured points to ensure a stable polynomial fit and a reliable derivative. Within this restricted band, the polynomial represents T(f) accurately with a moderate degree, free from both antenna contamination and endpoint oscillations, as shown in Figure 5.

### 3.2. Extracted Values

It is worth stressing that the differentiation in Equation (6) with respect to frequency assumes the permittivity ε to be locally constant, i.e., the medium is treated as non-dispersive. Under this assumption, the derivation is exact, and it remains accurate for weakly dispersive materials, whose permittivity varies negligibly over the narrow frequency window used for the local fit. The three materials characterized in this work (mineral oil, PMMA and dry sand) are non-dispersive to a very good approximation in a range of ±150 MHz centered on 1.568, so this condition is well satisfied. In the hypothetic case of materials presenting a highly dispersive response even if in a narrow frequency range, the frequency dependence of ε would have to be retained in the differentiation, so this constitutes a limitation of the present formulation.

Before extracting the permittivity, it was necessary to establish whether the system should be treated as lossless or lossy, since the two cases lead to different working equations: the lossless formulation of Equation (19) returns the real permittivity ε_r_ alone, while the lossy formulation from Equations (15) and (16) is required to retrieve the complex permittivity ε_r_ − jε_j_. To make this decision quantitatively, Equation (20), the lossless condition, T_r_·T_rd_ + T_j_·T_jd_ = 0, was evaluated for the three materials over the analysis band. If the left-hand side of Equation (20) is approximately zero, the negligible-loss assumption holds, and Equation (19) can be applied directly; if it deviates significantly from zero, the lossy formulation must be retained.

**Figure 6:**
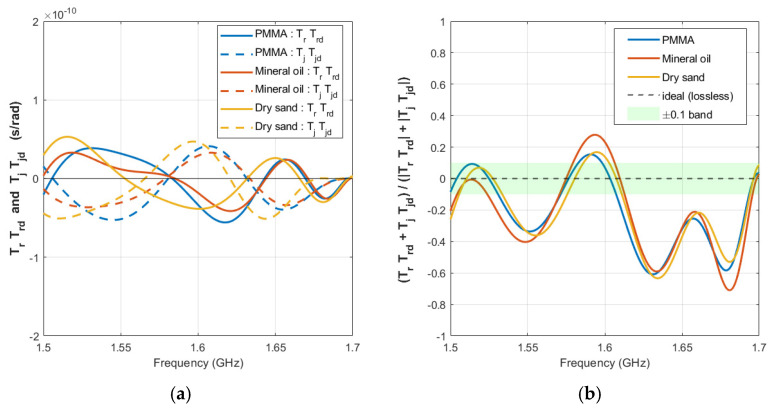
Left-hand side (LHS) of Equation (20): (**a**) raw data where real and imaginary parts show an opposite trend, leading to a sum close to zero; (**b**) normalized trend, where the denominator acts only as a reference scale (“ruler”).

Plotted in absolute terms (Figure 6a), Equation 20 yields very small numerical values (on the order of 10^−11^–10^−9^), making it impossible to state whether it is “close to zero”: a small number is only meaningful relative to the natural scale of the quantities involved. To obtain a dimensionless and interpretable indicator, the left-hand side (LHS) of Equation (20) is divided by the sum of the magnitudes of its two terms, (|T_r_·T_rd_| + |T_j_·T_jd_|). The resulting ratio is bounded between −1 and +1 (see Figure 6b): a value near 0 means the two terms cancel each other, so Equation (20) is satisfied and the material behaves as lossless, whereas a value near ±1 means one term dominates, the cancelation fails, and the lossless condition does not hold.

**Figure 7:**
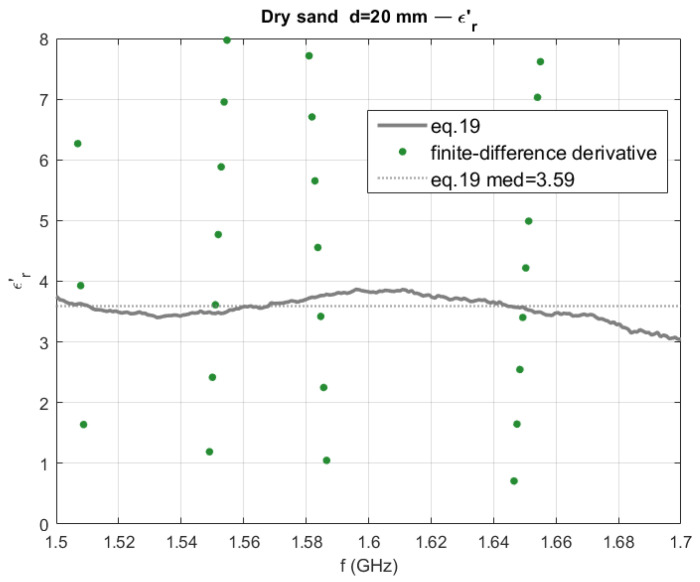
Permittivity calculated by direct numerical differentiation of the raw data and the fitted values (Equation (19))—dry sand.

Having verified that the negligible-loss condition reasonably is satisfied, the real permittivity can be retrieved directly from the lossless formulation, i.e., using Equation (19) and by direct numerical differentiation of raw data (Table 2). We calculated the permittivity both by the measured dot values and the fitted values (Figure 7): the scatter of the measured points around the fitted curve is precisely why a smooth fit is required rather than a direct numerical differentiation of the raw data; the derivative amplifies any point-to-point fluctuation, so computing dT/df by finite differences on the raw points magnifies the measurement noise and produces an unusable, strongly scattered permittivity.

As shown in Table 2, two different values of d (8 mm and 20 mm) were used, with very good agreement between the obtained dielectric constant values and literature data for both the thicknesses. The thickness of the MUT d is explicitly present in Equations (15), (16) and (19) but is also implicitly present in T and its derivatives. In fact, T is calculated from the measured values of the scattering parameters, which depend on d. For a high-loss material the attenuation across the sample grows with d and the transmitted signal eventually falls below the noise floor. This could be a problem for such a material, but it does not concern the MUTs analyzed in this article.

## 4. Conclusions

The derivative method was applied to free-space S-parameter measurements of three dielectric materials, after a preliminary check established that the negligible-loss assumption was appropriate and that the real permittivity could be retrieved through the lossless formulation.

An advantage of the approach concerns phase ambiguity. The derivative method involves only real quantities (T_r_, T_rd_, T_j_, T_jd_, |T|), whereas techniques that rely on the phase of the S_21_ signal to retrieve the dielectric permittivity are sensitive to it. Phase unwrapping yields a single, correct result only if the phase changes by less than π from one frequency step to the next, as discussed in theoretical and experimental papers on optical or microwave propagation [17,18]. If this change exceeds π at any point, the starting branch of the unwrapping may be chosen incorrectly. This produces a fixed 2πm phase offset, which in turn leads to an error in the retrieved ε_r_. As pointed out by Weir [4], the correct branch is determined by the sample length expressed in terms of the wavelength within the material; ambiguity is avoided if the sample is thin enough and the phase accumulated through it does not exceed one wavelength. It is worth noting that for high-permittivity materials or at high frequencies, this condition becomes more restrictive, and phase ambiguity turns into a practical concern.

The inferred real permittivity ε_r_ for the studied materials proved to be the robust output of the developed analytical method. PMMA (3.31) and dry sand (3.59) fall within the ranges reported in the literature (PMMA 2.6–3.4; dry sand 2.5–5.0 [14,15]). Mineral oil gives 1.85, slightly below the typical range for mineral oils (2.0–2.3 [16]), but internally consistent, since the corresponding refractive index n ≈ 1.35 is close to the expected n ≈ 1.39.

## Figures and Tables

**Table 1:** Experimental parameters.

Parameter	Symbol	Value	Unit
Antenna separation	D	15	cm
Frequency range	f	1.0–2.0	GHz
Frequency points	N	1601	—
Instrument	—	Keysight P9375A	—
MUT 1: Mineral oil	d	20	mm
MUT 2: PMMA	d	8	mm
MUT 3: Dry sand	d	20	mm

**Table 2:** Extracted permittivity in the narrowband ±150 MHz window centered at the S11 resonance.

Material	ε_r_ from Equation (19)	ε_r_ from Finite-Difference	ε_r_ by Literature
Dry sand (d = 20 mm)	3.59 ± 0.23	4.03 ± 18.83	2.5–5.0 [14]
PMMA (d = 8 mm)	3.31 ± 0.22	3.94 ± 12.46	2.6–3.4 [15]
Mineral oil (d = 20 mm)	1.85 ± 0.13	4.98 ± 18.84	2.0–2.3 [16]

## Data Availability

The data presented in this study are available on request from the corresponding author.
